# Environmental life cycle assessment of forestry biochar for low-carbon concrete: influence of biogenic carbon accounting

**DOI:** 10.1007/s11356-026-38335-1

**Published:** 2026-11-04

**Authors:** Supun Karunarathna, Chamila Gunasekara, David Law, Yulin Patrisia, Letiscia Xavier, Sujeeva Setunge

**Affiliations:** 1 Civil & Infrastructure Engineering, School of Engineering, RMIT University, Melbourne, Victoria 3000 Australia; 2 Vital Chemical, 170 Bellwood St, Darra, Queensland 4076 Australia

**Keywords:** Life cycle assessment, Carbon sequestration, Portable pyrolysis systems, Biochar, Biochar concrete, Carbon accounting methodologies, Circular economy

## Abstract

As global interest grows in innovative and sustainable uses of biomass, understanding the environmental implications of integrating timber-derived biochar into concrete is increasingly important. This study presents a cradle-to-gate life cycle assessment (LCA) of biochar production and its application as a 15% sand replacement in 32 MPa concrete, evaluating four portable biochar production systems, the 6050 Carbonator, Air Curtain Burner, Oregon Kiln, and Biochar Systems Incorporated, using feedstocks sourced from forest residues and plantation-derived timber. Two biogenic carbon accounting approaches were applied: the carbon–neutral approach and the recommendation approach. The results show that carbon accounting choices strongly influence Global Warming Potential (GWP) outcomes. Under the Carbon–Neutral approach, the 6050 Carbonator exhibited the lowest net GWP (–1.90 kg CO_2_-eq kg⁻^1^ biochar), while the corresponding biochar-modified concrete achieved an approximately 40% lower net GWP than conventional concrete when carbon sequestration was included. Under the Recommendation approach, which explicitly includes biogenic CO_2_ flows, the plantation-derived biochar concrete scenario achieved a net-negative GWP of –17.49 kg CO_2_-eq m⁻^3^ due to biogenic carbon uptake during forest growth. However, several non-GWP impact categories, including acidification, ozone depletion, and eutrophication, increased due to forestry operations and long-distance transportation, with transport contributing up to 78% of impacts in some categories. Overall, the study demonstrates the strong influence of biomass sourcing assumptions and biogenic carbon accounting methodology on LCA outcomes for biochar-based concrete systems. The findings highlight the potential of forestry biochar to support lower-carbon construction while emphasizing the importance of transparent system boundaries, transport logistics, and carbon accounting approaches in sustainability assessments.

**Supplementary Information:**

The online version contains supplementary material available at https://doi.org/10.1007/s11356-026-38335-1.

## Introduction

### Background

In recent years, pursuing environmental sustainability has become increasingly critical, particularly in the construction industry. The production of concrete, one of the most widely used construction materials globally (Al Khaffaf et al. 2025), poses significant environmental challenges due to the intensive use of natural resources and substantial carbon emissions (Patrisia et al. 2022). Addressing these issues requires a shift toward more sustainable construction practices, including the adoption of green concrete, a form of concrete in which traditional cement and aggregates are partially or fully replaced with waste-derived or natural alternative materials (Ahdal et al. 2022; Sivakrishna et al. 2020).

Green concrete offers a sustainable alternative to conventional concrete by reducing greenhouse gas emissions and improving resource efficiency. Studies show that incorporating locally available waste and alternative natural materials can deliver comparable or enhanced performance, along with longer service life and reduced maintenance needs (Ahdal et al. 2022; Sivakrishna et al. 2020). A major environmental concern in concrete production is the high demand for natural aggregates, which leads to resource depletion and ecological damage. In this context, the use of solid waste–derived aggregates (direct, recycled, or upcycled) provides a practical solution to conserve natural resources, improve waste management, and lower the embodied energy and carbon footprint of concrete (Gupta et al. 2022; Patrisia et al. 2025a).

Timber and wood waste represent a significant portion of global waste, with Australia alone generating approximately 2.4 million tonnes annually, of which only about 879 kilotons are recycled, while the rest is largely landfilled or used for energy recovery (Department of Climate Change Energy the Environment and Water 2022a; Karunarathna et al. 2025). The Australian forest industry produces large quantities of wood residues from harvesting and sawmill operations, including stumps, bark, tree crowns, and heads, much of which is typically left on-site to support forest regeneration (Australian Bureau of Agricultural and Resource Economics and Sciences 2018). However, unmanaged forest residues pose serious environmental risks, particularly by increasing the likelihood of catastrophic wildfires, which have devastating social and economic impacts (Limenih et al. 2024; Puettmann et al. 2020). Such risks are not limited to Australia; global examples, like the 2025 California wildfires, demonstrate the scale of potential damage, with estimated property losses ranging from $28 to $53.8 billion (Maccarthy and Richter 2025; Lloyd 2025).

Forest residues are often pile-burned to clear land for replanting, yet this practice contributes to air pollution, may degrade soil quality, and can inadvertently lead to uncontrolled fires if not properly managed (Puettmann et al. 2020; Oneil et al. 2017). Despite their abundance, the utilization of forest residues remains limited due to high logistical and machinery costs associated with collection and transport over large, dispersed areas. Controlled burning, though common, contributes to air pollution and adverse impacts on human health (Oneil et al. 2017).

Converting forest residues into value-added products such as biochar for construction applications offers a more sustainable alternative to residue management (Gupta et al. 2018; Puettmann et al. 2020). Among various production technologies, slow pyrolysis is the most commonly used method for timber and wood biochar conversion, as it is optimized for biochar yield rather than co-products like bio-oil or syngas, which are prioritized in other thermal conversion processes (Belletti et al. 2021). In recent years, mobile and portable pyrolysis systems have gained attention for enabling decentralized production and reducing transport-related impacts. However, biochar properties and environmental performance vary widely depending on production conditions, including pyrolysis technology, temperature, heating rate, pressure, feedstock type, and pre- and post-treatment processes (Ma et al. 2019; Karunarathna et al. 2025).

Biochar is widely recognized for its carbon sequestration potential and has been utilized in various sectors, including soil amendment, fuel, landscaping, biofilm carriers, and more recently as a construction material (Azzi et al. 2022). Biochar has been effectively used in concrete as a partial replacement for cement, sand, and as a filler (Belletti et al. 2021; Gupta et al. 2018; Liu et al. 2025; Roychand et al. 2023; Karunarathna et al. 2025), improving internal curing, reducing shrinkage, and enhancing sustainability. When used as a cement substitute, incorporating 1–5% biochar has been reported to increase compressive strength by up to 45% and reduce shrinkage by 25% (Ling et al. 2023; Gupta et al. 2020). As a sand replacement, biochar has also shown promising results: for instance, 3% soybean dregs biochar improved compressive and tensile strength by 12.32% and 16.42%, respectively (Liu et al. 2025). A 23% increase in 28-day compressive strength was reported when 20% of the sand was replaced with timber biochar (Roychand et al. 2023).

Recent studies have evaluated the environmental implications of biochar utilization in concrete and other urban applications, including consideration of biogenic carbon sequestration and carbon accounting approaches (Azzi et al. 2022; Chen et al. 2022). For example, Azzi et al. 2022 assessed multiple urban biochar applications, including tree planting systems, green roofs, landscaping soils, charcrete, and biofilm carriers, using broader urban-scale system boundaries and several biochar supply chains, whereas the present study specifically focuses on timber-derived biochar integrated into structural concrete as a partial sand replacement material. Chen et al. 2022 investigated biochar-integrated mortar and concrete block systems with emphasis on carbon sequestration and climate mitigation performance. However, despite increasing interest in biochar-integrated cementitious materials, limited studies have comparatively assessed the life cycle environmental performance of timber-based biochar used specifically as a partial sand replacement in concrete applications, particularly when produced using portable or mobile forestry biochar production systems. In addition, previous studies have generally focused on single production pathways or specific application scenarios, whereas the present study comparatively evaluates multiple forestry biochar production technologies and examines the influence of different biogenic carbon accounting approaches across the full pathway from biomass collection and biochar production to concrete application.

The majority of existing LCAs apply a carbon-neutrality assumption, under which biogenic CO_2_ uptake and release are assumed to balance over the biomass life cycle and are therefore excluded from GWP calculations (Puettmann et al. 2020; Sahoo et al. 2021). Consequently, the climate mitigation benefits associated with long-term biogenic carbon storage in biochar are not always consistently represented within reported GWP outcomes. Furthermore, many studies rely on a single carbon accounting framework without critically examining how alternative approaches may influence results and their interpretation. In contrast, LCAs in the concrete sector typically adopt the recommendation method, which includes all greenhouse gas emissions, both fossil and biogenic, in the GWP calculation, in line with ISO 14067 and ILCD guidelines (Renouf et al. 2016). This is particularly important for assessing biochar-based concrete, where biogenic CO_2_ emissions from biochar production and the potential for long-term carbon sequestration can significantly affect the net climate impact.

Such methodological divergence limits the comparability of LCA results across sectors. To address this issue, the present study employs both carbon–neutral and recommendation methodologies to evaluate the life cycle of biochar production and its integration into concrete. By comparing the outcomes of these two approaches, this research aims to highlight the influence of methodological choices on LCA results and support evidence-based decision-making in policy development and sustainable construction practices.

### Significance of research

This study provides a comparative environmental assessment of forestry-derived biochar integrated into concrete under different biomass sourcing and biogenic carbon accounting assumptions. By evaluating portable biochar production systems and timber biochar as a sand replacement material, the study provides practical insights into low-carbon concrete production, circular utilization of forestry residues, and long-term carbon sequestration within construction materials. The findings support industry and policy decision-making for sustainable construction and climate mitigation strategies.

## Methodology

### Goal and scope definition

**Fig. 1:**
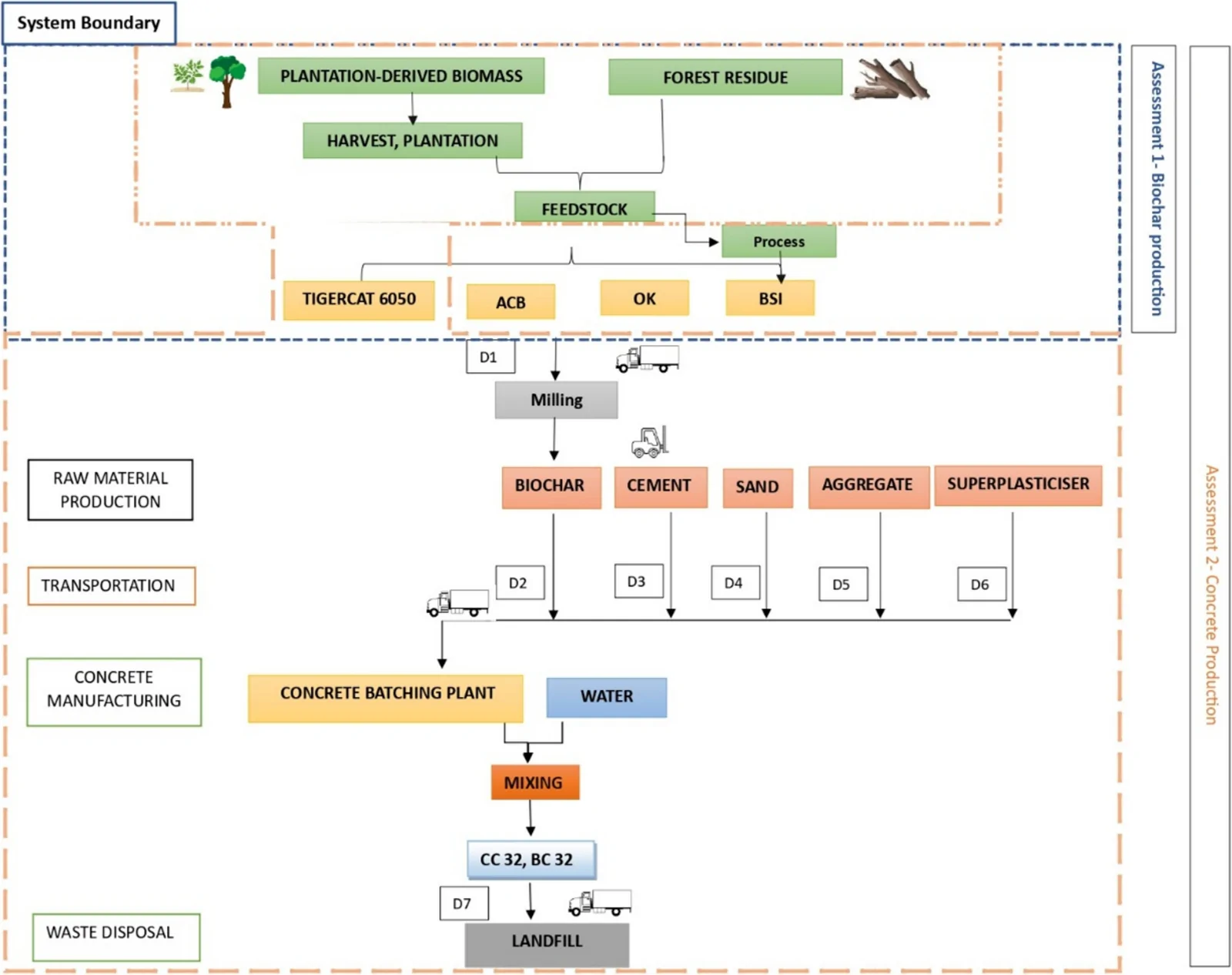
System boundaries

The primary goal of this study is to evaluate the environmental impacts associated with the use of timber biochar as a partial sand replacement in the production of 32 MPa concrete through a comparative LCA. In the biochar-modified concrete mix, 15% of the natural sand fraction by weight was substituted with timber-derived biochar. The analysis was divided into two distinct yet interconnected assessments: (i) biochar production, and (ii) concrete manufacturing, as illustrated in Fig. 1.

In Assessment 1, biochar production is modelled using a cradle-to-gate system boundary, encompassing all upstream processes from raw material acquisition to biochar preparation. This includes feedstock preprocessing and biochar production using four commonly adopted technologies in the industry: 6050 Carbonator, Air Curtain Burner (ACB), Oregon Kiln (OK), and Biochar Solutions Incorporated (BSI). The system boundary concluded at the point where biochar is ready for transport for milling, with particles smaller than 4 mm to be used to replace sand.

For both biomass sourcing assumptions, environmental impacts were normalized to the functional unit of 1 kg of produced biochar in Assessment 1, enabling consistent comparison of biochar production pathways despite differences in upstream system boundaries and biomass origin. Inventory calculations were initially based on biomass feedstock input and subsequently normalized to 1 kg of produced biochar using the corresponding biochar yield of each production system. Therefore, systems with lower biochar yield required greater feedstock input and associated upstream processing per functional unit of produced biochar.

In Assessment 2, concrete manufacturing was assessed using a cradle-to-gate perspective, covering the extraction, processing, and transport of materials including cement, aggregates, water, superplasticizer, and biochar. The system boundary ended at the concrete production stage, with concrete prepared for footpath application. Environmental impacts were normalized to the functional unit of 1 m^3^ of concrete based on the total material and process inputs associated with each concrete mix design. Biochar for the concrete assessment was selected based on the lowest net GWP under the carbon–neutral method in Assessment 1.

### Biochar production

#### Biomass

Timber-derived biomass is used as the biochar feedstock, following two commonly adopted LCA approaches for defining system boundaries and biogenic carbon treatment. In the first approach, biomass is considered a residual or by-product derived from forest residues, logging residues, or industrial wood processing streams. Under this assumption, upstream forestry activities such as plantation establishment and harvesting are excluded from the system boundary, as the biomass is not harvested specifically for biochar production (Puettmann et al. 2020; Sahoo et al. 2021; Azzi et al. 2019, 2022).

In the second approach, biomass is treated as a primary product, and the system boundary includes plantation establishment, forest growth, and harvesting processes, with biogenic CO_2_ uptake during tree growth explicitly represented (Rajabi Hamedani et al. 2019; Puettmann et al. 2020).

Accordingly, this study evaluates two biomass sourcing assumptions: (i) residual timber biomass and (ii) plantation-derived timber biomass. For the plantation-derived scenario, eucalyptus forestry datasets representing existing managed plantation systems under business-as-usual conditions were adopted, including fertilization, harvesting operations, and regrowth cycles over a 14-year rotation period with two harvesting events. However, direct land-use change emissions associated with conversion of natural vegetation into plantation land were not explicitly included. Both biomass sourcing assumptions were evaluated using the carbon–neutral and recommendation biogenic carbon accounting methods, as illustrated in Fig. 1.

#### Biochar production technologies

Four biochar production pathways were evaluated: the Tigercat 6050 Carbonator, Air Curtain Burner, Oregon Kiln, and the Biochar Solutions Incorporated gasifier system. These technologies represent different operational scales and thermochemical conversion approaches, including flame-cap pyrolysis and continuous gasification systems. The Tigercat 6050 is a mobile flame-cap pyrolysis unit designed for in-field carbonization of woody residues without feedstock preprocessing. The ACB and OK systems are batch-operated flame-carbonization technologies used for biomass reduction and biochar production from forest residues (Sahoo et al. 2021; Cornelissen et al. 2016; Zaramí 2021). The BSI system is a continuous down-draft gasifier designed for decentralized biochar production from chipped woody biomass (Biochar Solutions Inc. 2025). Detailed descriptions of the technologies and operational parameters are provided in the Supplementary Information (Section S1).

### Carbon sequestration potential

Biochar has traditionally been utilized in soils to enhance carbon sequestration, remediation, and fertility (Lehmann et al. 2006; Ahmad et al. 2014; Biederman and Harpole 2012; Wang et al. 2016). Regardless of application, biochar contains both labile and recalcitrant carbon fractions, the latter of which can remain stable in the environment for centuries (Singh et al. 2012). Stability is influenced by factors such as feedstock type, pyrolysis temperature, and biochar properties, particularly the H/C_org_ molar ratio, a key indicator of carbonization (Sahoo et al. 2021). Biochar with H/C_org_ ≤ 0.7 are considered stable and suitable for long-term carbon storage, even in non-soil applications (puro.earth 2024).

No peer-reviewed methodologies currently exist for quantifying long-term carbon storage in non-soil applications such as concrete; this study conservatively applies models originally developed for soil environments. Specifically, the Biochar Methodology – Edition 2022, Version 3 by Puro.earth is used, which estimates carbon sequestration using the H/Cₒᵣ_g_ molar ratio and regional temperature parameters (puro.earth 2024). While these models are tailored for soil use, their application to concrete is considered conservative and reasonable, as biochar embedded in mineral matrices like concrete is expected to degrade even more slowly, thus offering comparable or greater long-term stability. The total amount of sequestered carbon (${E}_{\mathrm{s}\mathrm{t}\mathrm{o}\mathrm{r}\mathrm{e}\mathrm{d}}$) is calculated as:

1 $${{{E}}}_{{{s}}{{t}}{{o}}{{r}}{{e}}{{d}}} ={{{Q}}}_{{{b}}{{i}}{{o}}{{c}}{{h}}{{a}}{{r}}} \times {{{C}}}_{{{o}}{{r}}{{g}}} \times {F_p^{TH,T_{s}}} \times \frac{44}{12}$$

where ${\mathrm{Q}}_{\mathrm{b}\mathrm{i}\mathrm{o}\mathrm{c}\mathrm{h}\mathrm{a}\mathrm{r}}$ = dry biochar mass (t), ${\mathrm{C}}_{\mathrm{o}\mathrm{r}\mathrm{g}}$ = organic carbon content (dry wt.%), $F_p^{TH,T_{s}}$ = permanence factor over 100 years, 44/12 = carbon to CO_2_ conversion factor.

The permanence factor, based on the H/Cₒᵣ_g_ molar ratio and regional soil temperature (assumed 18.6 °C for Melbourne at 10 m depth) (Narsilio et al. 2014), is used to estimate long-term carbon storage. Though developed for soils, it is conservatively applied here, as concrete-embedded biochar may offer even greater stability. In this study, the permanence factor is used to calculate the amount of stable carbon retained in concrete over time, which is then included in the net GWP results and informs the potential for carbon credit allocation and sustainability assessment. The H % used for this calculation is sourced from published literature on wood-derived biochar with comparable fixed carbon content (Senadheera et al. 2023; Rehrah et al. 2016).

### Concrete manufacturing process

The functional unit for the concrete manufacturing stage is defined as 1 m^3^ of 32 MPa concrete. A consistent mix design was applied to both the control concrete (CC32) and the biochar-modified concrete (BC32), with 15% timber biochar used as a sand replacement by weight in the BC32 mix. This proportion was selected to evaluate environmental impacts rather than to represent a strength-optimized mix. Although experimental mechanical validation was not conducted within the present study, the selected replacement level was based on literature-reported performance of timber biochar concrete (Roychand et al. 2023), where compressive strengths comparable to or higher than the control concrete have been reported within the selected replacement range. Furthermore, the intended application considered in this study was pavement and footpath concrete, where the required compressive strength is generally above 20 MPa. Therefore, a volumetric functional unit of 1 m^3^ of concrete was considered appropriate for comparing environmental impacts within the same intended application context, rather than using a strength-normalized functional unit. The mixing proportions for both the control concrete and the biochar-modified concrete are presented in Table 1. The same admixture dosage was used for the BC32 mix and the control concrete to maintain comparable workability and achieve the target slump (60–120 mm), consistent with previous studies on biochar used as a sand replacement (Roychand et al. 2023; Hansen and Berglund 2023; Liu et al. 2025). To avoid excessive water absorption and maintain the intended water-to-cement ratio, the biochar was pre-soaked prior to mixing, following established procedures in the literature (Gupta and Kua 2018).

**Table 1:** Mix design for CC32 and BC32 (kg/m^3^)

Raw materials	Unit	Type of concrete	
		CC32	BC32
Water	kg	155	155
Water for biochar saturates (water absorption 1.5 g/g)	kg	-	143.55
Cement	kg	360	360
Sand (oven dried)	kg	638	542
Coarse Agg max 10 mm (SSD)	kg	821	821
Coarse Agg max 7 mm (SSD)	kg	411	411
Optimal biochar dosage	%	-	15
Biochar	kg	-	95.7
Admixture	kg	1.8	1.8

### Life cycle inventory

Data for the LCA were compiled from primary measurements, technical specifications, published literature, and databases available within the SimaPro 10.2.01 software. The modelling was supported by Ecoinvent v3.8 (2021) and AusLCI v1.42 (2023), using background inventory datasets based on the cut-off system model. A summary of key input data is presented in Supplementary Information (Table S1).

Foreground biomass sourcing assumptions followed the system boundary definitions described in the “Biomass” section. Residual timber biomass was treated as a residual by-product, whereas plantation-derived biomass was modelled using eucalyptus forestry datasets representing existing managed plantation systems. Direct land-use change emissions associated with initial land conversion were not included.

Feedstock requirements per tonne of biochar vary due to differences in yield efficiency. The BSI system consumes diesel for feedstock preparation, transport, and electricity generation. Energy inputs for all systems, along with logistical operations such as size reduction, loading, and transport, are outlined in Table S2.

All equipment is mobile, enabling on-site biochar production and minimizing feedstock transport emissions. The 6050 and ACB process similar feedstocks, while the OK requires smaller material, produced with chainsaws. The BSI system needs chipped and ground biomass, requiring additional preprocessing. Propane was used as the startup fuel for the pyrolysis process in the ACB, OK, and BSI systems (Puettmann et al. 2020; Sahoo et al. 2021). In the Tigercat 6050 Carbonator, diesel-soaked rags on poles were used for ignition, consuming approximately 37.85 L of diesel. This fuel is used only during the initial ignition phase (Air Burners Inc. n.d.).

Due to the lack of direct emissions data and diesel usage for initial ignition for the 6050 Carbonator, this study applies EPA data from air curtain incinerators as a worst-case proxy, as those systems fully combust biomass to ash. In reality, the 6050 retains biochar and thus likely results in lower actual emissions (Tigercat International Inc. 2020). All energy and emissions data relevant to biochar production are compiled in Table 2.

**Table 2:** Input and output data per ton of the Biochar Production process

Input resources	Units	Tigercat 6050	OK	ACB	BSI
Input					
Feedstock	kg	13,333	5000	5000	6781
Biochar yield	%	7.5	20	20	16
Operating temperature	°C	820–1370	680–750	680–750	Lower-temperature gasification
Process type		Flame-curtain/flame-cap pyrolysis	Flame-cap kiln	Flame-curtain pyrolysis	Downdraft gasification
Propane	L		1,020	441	1,727
Diesel	kg	10.85	-	-	91.5
Water for quenching	L	661	2000	2000	-
Output					
Fixed carbon	%	85	76	89	65
Emission					
Carbon dioxide, biogenic	kg	9.83E+03	3.90E+03	3.90E+03	1.29E+04
Carbon monoxide, biogenic	kg	1.73E+01	1.30E+01	1.30E+01	3.56E+00
Methane, biogenic	kg	2.628E+01	1.30E+01	1.30E+01	7.80E−01
Nitrogen monoxides	kg		7.00E−01	7.00E−01	
Nitrogen oxides	kg	1.83E+00	7.20E−01	7.20E−01	1.00E+01
Nitrous oxide	kg	9.31E−1			
Particulates, <10 um	kg	4.41E−02	6.40E+00	6.40E+00	7.05E+00
Particulates, <2.5 um	kg	2.06E−02			6.21E−02
Propane	kg				2.14E+00
Sulphur dioxide	kg	1.51E+00			1.78E−01
Total hydrocarbons (THC)	kg	6.41E−01			
Carbon sequestration	ton CO_2_ -eq	2.848	1.716	2.999	0.889

Although slow pyrolysis produces co-products such as syngas and bio-oil, these were assumed to be internally combusted to meet process energy demands. No separate allocation of environmental burdens to co-products was applied, and biochar was treated as the primary functional output.

Following production, the biochar is transported approximately 7 km to a nearby milling facility located near the forest site. The associated milling activities, including loading, unloading, and electricity/fuel use, are summarized in Table S3, based on data collected from production reports. In the base-case scenario considered in this study, the produced biochar is transported from Queensland to the concrete mixing site in Victoria, over an approximate distance of 2058 km. Naphthalene-based superplasticizers are used for both CC32 and BC32 concrete mixes for concrete production. Transport of raw materials, concrete, and construction waste is modelled via 16–28 tonne diesel trucks, using fleet-average values. A ready-mix concrete plant located 7.7 km from the construction site in outer Melbourne, Victoria, Australia, is considered. All transport distances are based on estimates from Google Maps (Table 3). Energy consumption during concrete production follows AusLCI databases (Sharma and Grant 2015; Patrisia et al. 2022).

**Table 3:** Transport distance

Route IDs	Distance (km)
D1	7
D2	2058
D3	35.9
D4	180
D5	180
D6	61.2
D7	11.8

### Life Cycle Impact Assessment (LCIA)

LCIA was conducted using the Australian Life Cycle Assessment Society (ALCAS) Impact Assessment Method (version 2.05), which converts life cycle inventory data into midpoint environmental impact categories for consistent comparison across systems.

Global Warming Potential (GWP100) was evaluated using two biogenic carbon accounting approaches defined within the ALCAS framework and aligned with ISO/TS 14067 and PAS 2050: the Carbon Neutral method and the Recommendation method.

Under the Carbon Neutral approach, biogenic CO_2_ emissions released during biochar production are excluded from GWP calculations based on the assumption that these emissions are balanced by CO_2_ uptake during biomass growth. Fossil-derived emissions associated with energy use are fully accounted for.

The Recommendation approach includes both fossil and biogenic CO_2_ emissions in the GWP calculation, providing a complete representation of carbon flows to and from the atmosphere. This method is applied to explicitly capture biogenic emissions associated with biomass growth, processing, and biochar production, particularly when plantation-derived biomass and forest activities are included within the system boundary.

For both accounting approaches, the net impact was determined by subtracting the carbon sequestration capacity associated with stable carbon retained in the biochar from the total GWP, reflecting the long-term carbon storage potential of biochar over the selected assessment period. This approach is commonly adopted in biochar LCA studies, particularly where residual biomass is used as feedstock (Tigercat International Inc. 2020; Sahoo et al. 2021; Puettmann et al. 2020; Azzi et al. 2022). The two frameworks were applied to both residual and plantation-derived biomass scenarios to evaluate the influence of biomass sourcing assumptions and carbon accounting methodology on GWP results.

The assessed midpoint impact categories include GWP100, Abiotic Depletion Potential (ADP), Ozone Depletion Potential (ODP), Photochemical Oxidation Potential (POP), Acidification Potential (AP), Eutrophication Potential (EP), Particulate Matter Formation (PM_2.5_), Human Toxicity Potential (HTP), Freshwater Aquatic Ecotoxicity Potential (FAETP), Ionizing Radiation – Human Health (HH), and Water Scarcity (Renouf et al. 2016).

## Results

### Assessment 1: Biochar production

Biochar production from residual wood was assessed using the Recommendation and Carbon Neutral LCIA method (Table 4). Only GWP differs between the Recommendation and Carbon–Neutral methods, while all other impact categories remain unchanged.

**Table 4:** Residual wood biochar production process environmental impact assessment per kg of biochar production

	Production method				
		6050	ACB	OK	BSI
Impact Category	Unit	Value			
Global warming (GWP100a) Carbon neutral method	kg CO_2_ -eq	9.45E−01	1.22E+00	2.43E+00	4.01E+00
Global warming (GWP100a) Recommendation method	kg CO_2_ -eq	1.08E+01	5.12E+00	6.33E+00	1.69E+01
Abiotic depletion (elem., econ. reserve)	kg SB -eq	3.63E−06	4.52E−05	1.04E−04	1.90E−04
Abiotic depletion (Fossil fuels)	MJ NCV	6.86E−02	7.15E−01	1.64E+00	2.92E+00
Ozone layer depletion (ODP)	kg CFC_-11_ -eq	3.55E−11	1.94E−10	4.43E−10	8.70E−10
Photochemical oxidation	kg C_2_H_4_ -eq	6.40E−04	6.25E−04	8.91E−04	1.31E−03
Acidification	kg SO_2_ -eq	4.49E−04	1.27E−03	2.89E−03	7.31E−03
Eutrophication	kg PO_4--_ -eq	3.63E−04	3.74E−04	8.56E−04	2.02E−03
Particulate matter	kg PM_2.5_	1.10E−04	1.80E−03	2.23E−03	3.41E−03
Human toxicity, cancer	CTUh	3.15E−12	3.59E−11	8.32E−11	1.53E−10
Human toxicity, non-cancer	CTUh	1.97E−12	1.50E−11	3.46E−11	5.94E−11
Freshwater ecotoxicity	CTUe	1.29E−04	1.37E−03	3.22E−03	5.45E−03
Ionizing radiation HH	kBq U_235_ -eq	4.01E−04	6.43E−03	1.48E−02	2.63E−02
Water Scarcity	m^3^ eq	−2.65E−04	−2.55E−01	−5.93E−01	−1.01E+00

#### Global warming potential

GWP represents the relative climate impact of greenhouse gas emissions over a 100-year time horizon, expressed as kg CO_2_-equivalent. Table 5 reports the GWP & net impact results for biochar production under both the Recommendation and Carbon–Neutral accounting approaches. Under the Recommendation method, the BSI system exhibits the highest production-stage GWP (16.9 kg CO_2_-eq kg⁻^1^ biochar), followed by the Tigercat 6050 (10.8 kg CO_2_-eq kg⁻^1^). Under the Carbon–Neutral approach, the Tigercat 6050 shows the lowest GWP, followed closely by the ACB system.

**Table 5:** Biochar production: GWP impact assessment

		6050	ACB	OK	BSI
		kg CO_2_ -eq./kg of biochar			
Sequestration capacity		2.85E+00	3.00E+00	1.72E+00	8.89E−01
Recommendation	GWP	1.08E+01	5.12E+00	6.33E+00	1.69E+01
	Net Impact	7.93E+00	2.12E+00	4.62E+00	1.60E+01
Carbon neutral	GWP	9.45E−01	1.22E+00	2.43E+00	4.01E+00
	Net Impact	−1.90E+00	−1.78E+00	7.15E−01	3.12E+00

2 $$Net\;impact = GWP - Sequestration\;Capacity$$

Carbon sequestration capacity, calculated using Eq. 1, reflects the amount of carbon retained in stable biochar. The ACB system demonstrates the highest sequestration potential (−3.0 kg CO_2_-eq kg⁻^1^ biochar), attributable to its higher fixed carbon content (Table 2). When sequestration is accounted for, the net climate impacts of the ACB and OK systems are low positive under the Recommendation method, while the Tigercat 6050 remains positive. Under the Carbon–Neutral assumption, ACB and Tigercat 6050 exhibit net-negative impacts, whereas the OK system becomes near-neutral and the BSI system remains positive.

These results are consistent with previous findings of biochar production in the literature. Puettmann et al. (2020) using the carbon–neutral concept in their analysis of biochar systems, reported a GWP range of 0.5 to +2.5 kg CO_2_-eq per kg of biochar, depending on the production method. Azzi et al. (2022) evaluated biochar produced from wood pellets, garden waste, and logging residues via fossil fuels slow pyrolysis in both syngas-heated and electricity-heated reactors, and reported GWP values ranging from +0.5 to +6 kg CO_2_-eq per kg of biochar. In contrast, Rajabi Hamedani et al. (2019) reported a net GWP impact of − 2.089 kg CO_2_-eq per kg biochar from willow wood chips via slow pyrolysis, using a net carbon balance approach that accounts for biogenic CO_2_ uptake during biomass growth alongside production emissions. This highlights the influence of feedstock type, system boundaries, and biogenic carbon treatment on reported GWP results.

#### Impact of plantation and forest harvesting

The inclusion of the wood plantation and harvesting in the LCA significantly affects the GWP of biochar production due to the biogenic CO_2_ uptake during tree growth. Australian forests alone are estimated to store 10.5 billion tonnes of carbon, with forest vegetation removing approximately 38.5 billion tonnes of CO_2_ from the atmosphere, equivalent to 77 times Australia’s annual net GHG emissions (Forest and Wood Products Australia 2023). This highlights the important role of tree cultivation in climate mitigation. When forests are sustainably managed and harvest residues are utilized as biochar instead of being left to decay or burned in piles, the GHG reduction potential increases significantly. The CO_2_ absorbed during tree growth helps offset emissions from harvesting, processing, and transport, particularly when compared to emissions from natural decomposition or uncontrolled burning (Forest and Wood Products Australia 2023; Azzi et al. 2019).

**Fig. 2:**
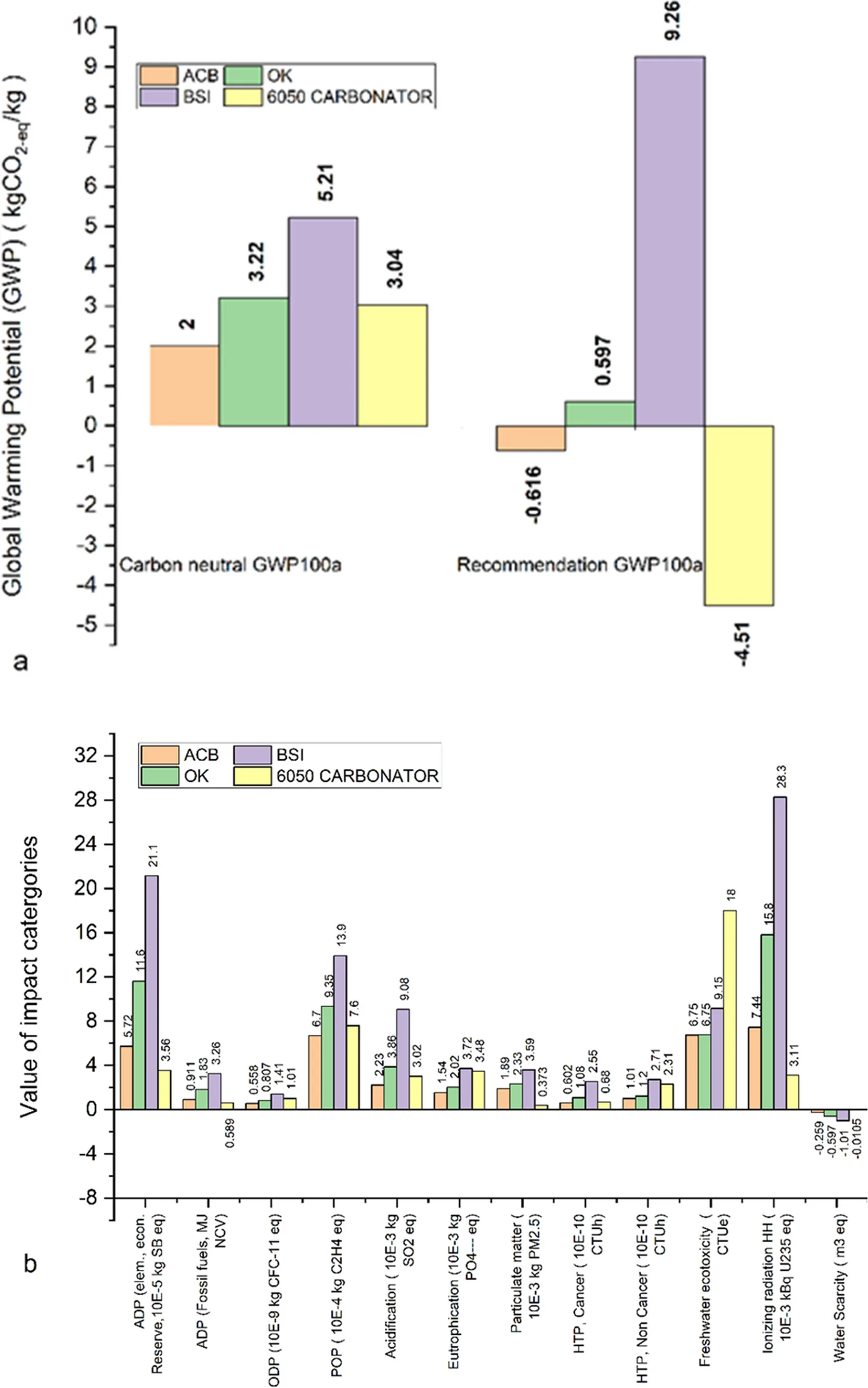
Environmental impacts of biochar production under the plantation-based system boundary: (**a**) GWP impacts under carbon–neutral and recommendation methods; and (**b**) non-GWP environmental impact categories

Figure 2 presents the (a) GWP (b) Non-GWP environmental impact assessment of biochar production under a plantation-based system boundary, covering processes from plantation establishment and harvesting through to biochar production. Under the Recommendation method, the inclusion of biogenic CO_2_ uptake during forest growth offsets emissions associated with biochar production, leading to reduced GWP. As a result, negative GWP values are observed for ACB (−0.616 kg CO_2_-eq/kg biochar) and Tigercat 6050 (−4.513 kg CO_2_-eq/kg biochar), while the OK and BSI systems exhibit lower but still positive GWP values (0.597 and 9.258 kg CO_2_-eq/kg biochar, respectively).

**Fig. 3:**
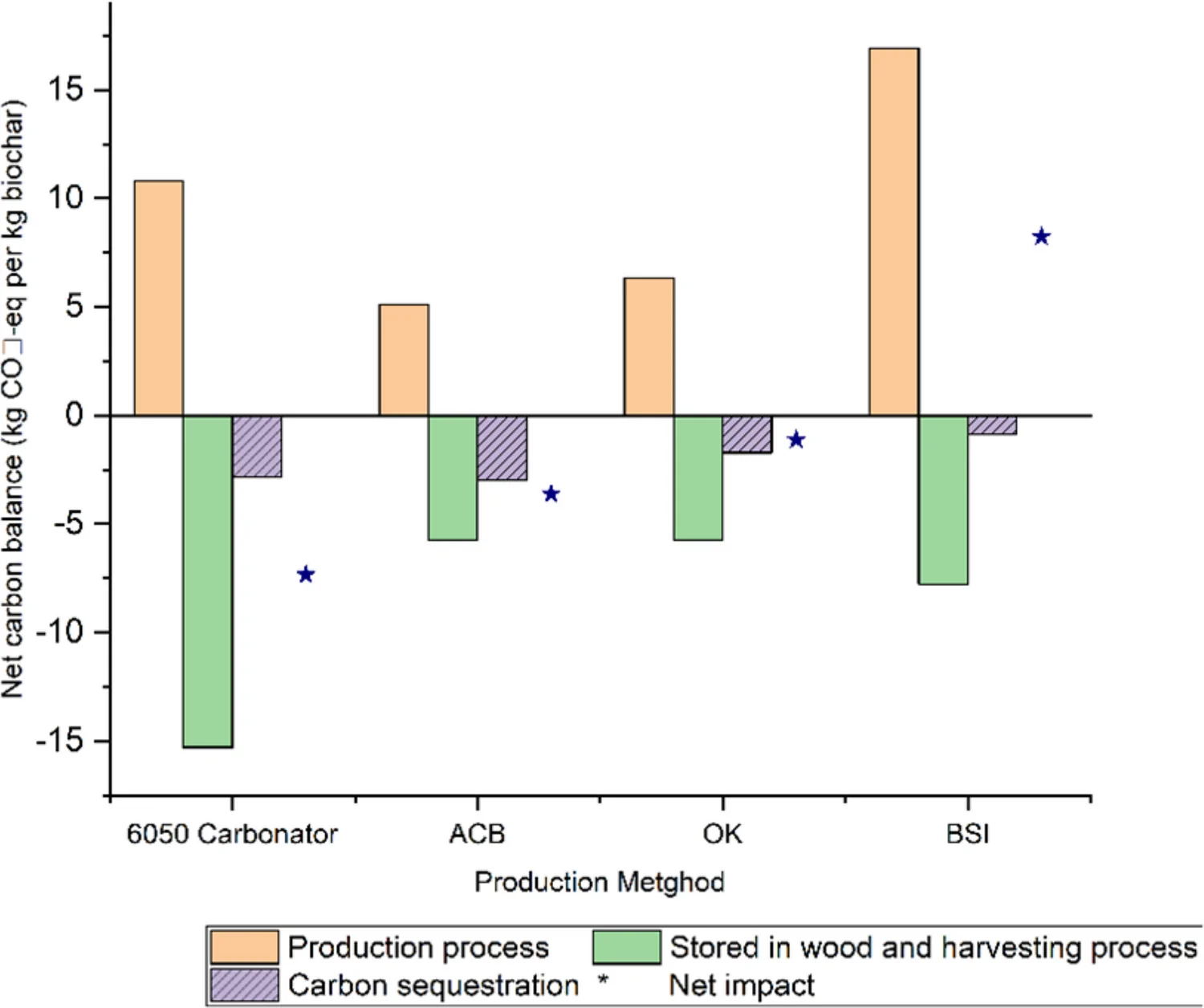
Net carbon balance of different biochar production systems, showing contributions from production emissions, biogenic CO_2_ uptake during biomass growth and harvesting, and long-term carbon sequestration in biochar

Figure 3 extends the analysis by presenting the net carbon balance, in which long-term carbon storage in biochar is accounted for separately from the GWP indicator. Biochar carbon sequestration is treated as an additional credit rather than being embedded within GWP. While this approach is debated, as the stored carbon ultimately originates from atmospheric CO_2_ uptake during biomass growth, it remains widely used to illustrate the long-term mitigation potential of biochar systems. When this carbon storage is included, the net carbon balance becomes −3.614, −1.119, 8.369, and −7.362 kg CO_2_-eq/kg biochar for ACB, OK, BSI, and Tigercat 6050, respectively, indicating that several production systems can act as net carbon sinks when both upstream biogenic uptake and stable carbon storage are considered.

The CO_2_ sequestration attributed to forest growth (stored in wood and the harvesting process) is based on a value of −1.147 kg CO_2_-eq per kg of harvested wood in this study. Even higher sequestration rates, ranging from −1.6 to −2.0 kg CO_2_-eq per kg, have been reported (Puettmann et al. 2020) although their analysis does not include emissions from plantation establishment and harvesting operations. It is important to note that while GWP is reduced, including forest growth and harvesting processes results in increased impacts in other environmental categories, such as ADP, ODP, and AP, except Water Scarcity, which shows a further reduction in impact across all four biochar.

Forest residue, including defective tree stems, bark, branches, and leaves, generated during harvesting and timber processing, is often left in forests or disposed of in landfills. This can lead to the emission of toxic gases (Aman et al. 2022) and gradual biological decomposition. This decay process emits GHGs, primarily CO_2_ under aerobic conditions and methane under anaerobic conditions. The CH_4_ produced is included in the GWP assessment due to its high GWP (Azzi et al. 2022; Forest and Wood Products Australia 2023; Garvie et al. 2021).

Using the methodology described in the National Inventory Report 2022 (Australia) (Department of Climate Change Energy the Environment and Water 2022b) and the IPCC 2006 Tier 2 First Order Decay (FOD) model, the parameters in Table S4 were used to estimate emissions from forest residue decomposition under anaerobic conditions: Based on this model, methane emissions from 1 kg of wood residue were calculated as

3 $$CH_4\;emitted=1\;kg\;wood\times DOC \times DOC_f \times MCF \times F \times 16/12 \times (1-OF)=0.024\; {kg}\; {{CH}_4}$$

0.672 kg CO_2_−eq (0.024 × 28) and Carbon Dioxide Emission (CO_2_ = 1 × 0.4 × 0.1 × 1 × 44∕12) 0.147 kg CO_2_.

#### Environmental impact indicators

Additional environmental indicators including abiotic depletion potential (fossil fuels), particulate matter formation (PM_2.5_), and ozone depletion potential (United States Environmental Protection Agency 2025) were also evaluated. Detailed results and discussion of these indicators are provided in the Supplementary Information (Section S3.1).

### Assessment 2: concrete manufacturing

**Fig. 4:**
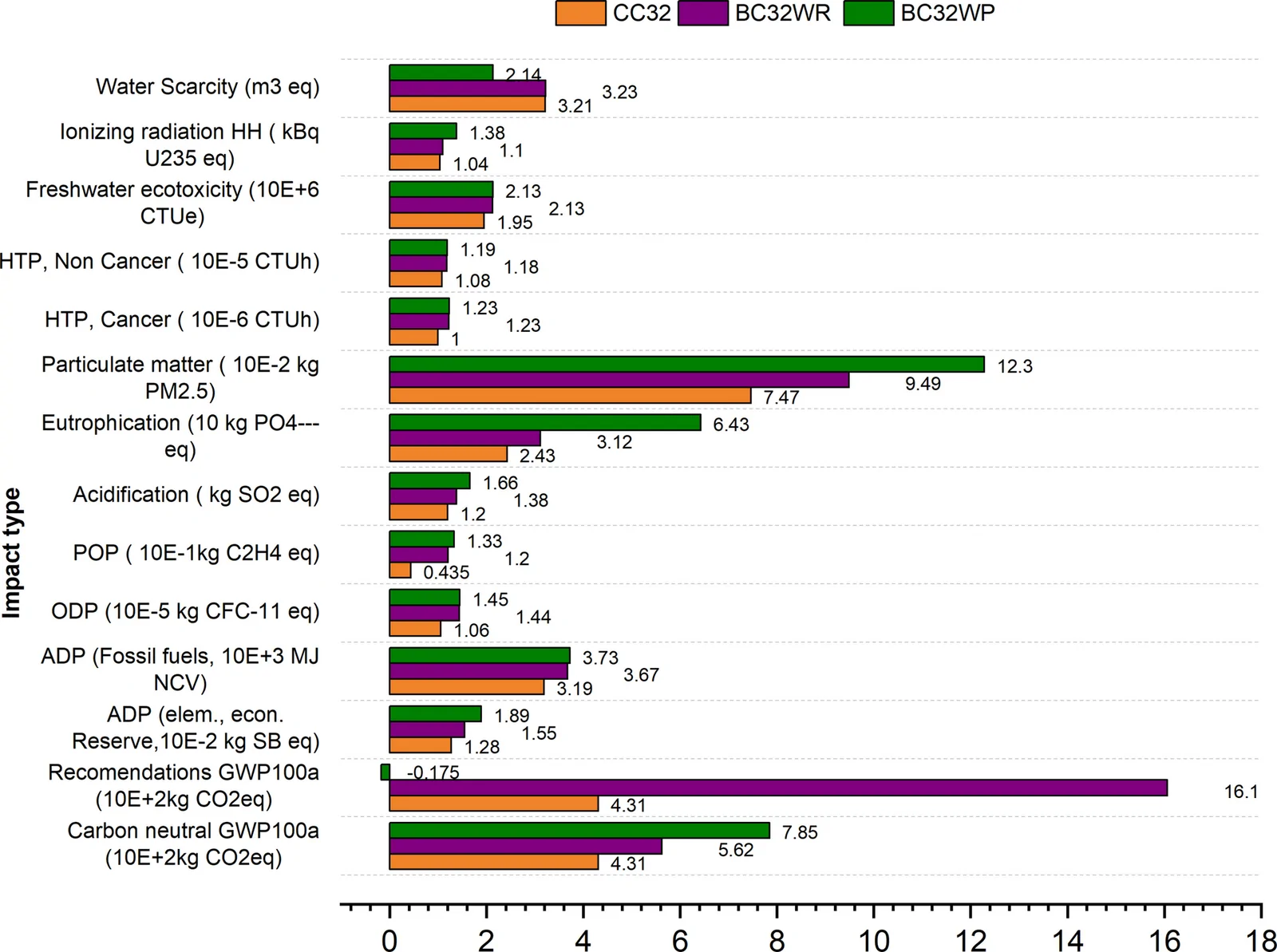
Environmental impacts of concrete manufacturing for CC32, BC32WR, and BC32WP representing different system boundaries. Results per 1 m^3^ of concrete

Based on the lowest net GWP impact observed under the carbon–neutral method, the 6050 Carbonator was selected as the preferred biochar production method for integration into the concrete manufacturing phase. The environmental impacts of the control concrete (CC32) and biochar-modified concrete (BC32) with 15% sand replacement are presented in Fig. 4.

Two BC32 scenarios were analyzed to represent different biochar supply pathways: (i) biochar derived from wood residues (BC32WR), where upstream forestry processes are excluded, and (ii) biochar derived from plantation-grown wood (BC32WP), where forestry operations are included. These scenarios are presented comparatively to evaluate the influence of feedstock origin and system boundary assumptions on environmental performance, rather than as directly equivalent systems.

Across most impact categories, both BC32 scenarios show higher environmental impacts compared to the control mix. However, in the GWP100a category under the Recommendation method, the BC32WP scenario demonstrates a climate benefit, primarily due to the biogenic carbon uptake associated with plantation-grown biomass, despite the analysis not accounting for carbon sequestration from the biochar itself.

#### Global warming potential

Table 6 presents the 100-year GWP results for the three concrete mixes, CC32, BC32WR, and BC32WP, evaluated under the two carbon accounting approaches. All results are expressed per functional unit of 1 m^3^ of concrete, ensuring consistency in comparison across scenarios despite differences in upstream system boundaries and biomass sourcing assumptions.

**Table 6:** Global warming impact (100-year GWP) of concrete production for CC32, BC32WR, and BC32WP under two carbon accounting approaches; results per 1 m^3^ concrete

		Control	BC32WP	BC32WR
		kg CO_2_ eq./m^3^ concrete		
Recommendation	GWP	431.01	−17.49	1606.57
	Net Impact		−320.17	1303.89
Carbon neutral	GWP	431.06	784.63	562.42
	Net Impact		481.95	259.74

The control sample shows the 431.01 kg CO_2_-eq/m^3^ value (431.01/32 = 13.5 kg CO_2_-eq/MPa), consistent with previous literature where the GWP of PC concrete was between 7.72 and 19 kg CO_2_-eq/MPa (Bajpai et al. 2020; Patrisia et al. 2022).

For the BC32WP, the Recommendation method results in a negative GWP (−17.49 kg CO_2_-eq/m^3^). This occurs because biogenic CO_2_ uptake during forest growth is explicitly included within the system boundary and offsets emissions from biochar production and concrete manufacturing. To avoid double-counting, no additional carbon sequestration credit is applied in this case as mentioned above.

For the BC32WR, excluding forest growth from the system boundary results in higher apparent climate impacts compared to the control concrete. Under the Recommendation method, emissions associated with biochar production and concrete manufacturing dominate the GWP, as no biogenic CO_2_ uptake is credited. Although long-term carbon storage in biochar reduces the net impact, the overall climate performance remains unfavourable relative to the control. Under the Carbon–Neutral method, the exclusion of biogenic CO_2_ flows leads to a lower GWP, with differences primarily driven by fossil energy use and material substitutions. In this case, the application of carbon sequestration credits substantially improves the net climate impact, reducing 40% compared to CC32, highlighting the sensitivity of BC32WR results to carbon accounting assumptions and the treatment of long-term carbon storage.

These findings align with Azzi et al. (2022), who assessed the climate impact of biochar-incorporated (charcrete) tiles (40 × 40 × 4 cm) and found that GWP was 62–130% higher than conventional concrete when biochar carbon sink was not considered. The studies also highlighted the role of supply chain variability, as emissions differed based on feedstocks such as wood pellets, garden waste, logging residues, willow woodchip biochar, and biochar-to-cement mass ratios ranging from 0.15 to 1.

**Fig. 5:**
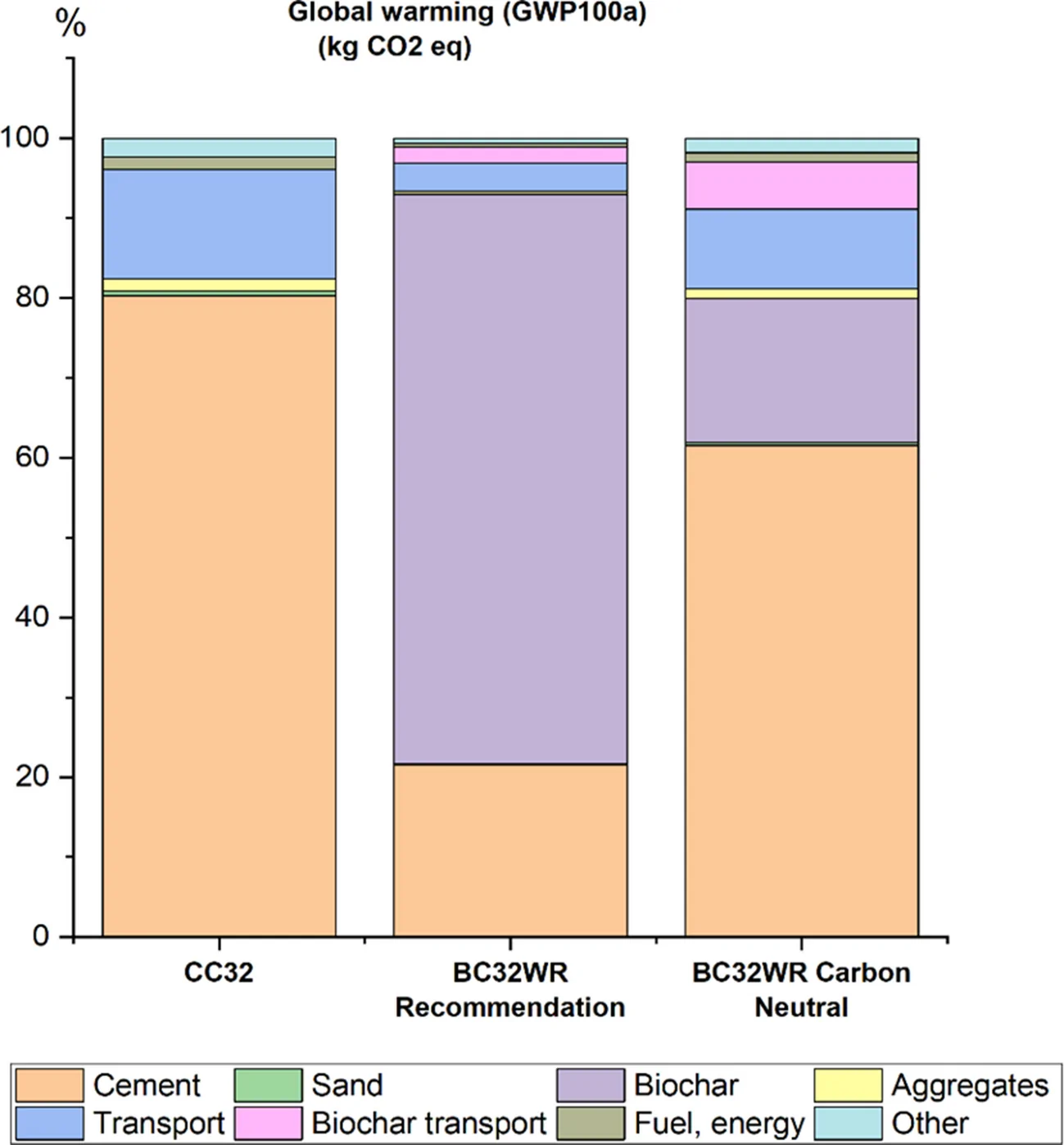
Contribution of materials, transport, and energy sources to total GWP for CC32 and BC32WR

According to the carbon mapping approach described by Yu et al. (2017) embodied emissions in construction materials such as cement and aggregates can be traced back to their industrial sources. This enables a more comprehensive assessment of the emissions burden associated with biochar-based products like BC32. In conventional concrete production, cement manufacturing remains the dominant contributor to GWP, accounting for over 80% of the total emissions in the control mix Fig. 5, consistent with previous findings highlighting cement’s major contribution to concrete emissions (Patrisia et al. 2022). Although sand is used in larger quantities by mass than cement, it contributes only 0.6%, while gravel contributes 1.52% to the total GWP, highlighting their comparatively minimal impact. Transportation-related emissions account for 13.7%.

In contrast, for the biochar concrete mix (BC32WR) assessed via the Recommendation method, the primary contributor to GWP shifts to biochar production, which accounts for 71.3% of total emissions. The contribution from cement is significantly reduced to 21.5%, and sand drops to just 0.14%. However, when analyzed under the Carbon Neutral concept, the impact of biochar production decreases substantially to 18%, while cement rises to 61.5%. Under this approach, sand and gravel account for 0.39% and 1.1%, respectively.

Transportation emissions also vary across scenarios. In the control mix, transport accounts for approximately 13.7% (58.9 kg CO_2_-eq) of total GWP. In the BC32WR mix, transport contributes 3.48% under the Recommendation method and 10% under the Carbon Neutral method, excluding the impact of biochar transport. Notably, biochar transportation alone contributes 33.19 kg CO_2_-eq, representing 2.07% and 5.9% of total GWP under the respective methods.

In this study, biochar is transported from Queensland to the concrete mixing site in Victoria, representing a long-distance transport scenario. Given the availability of forest resources in Victoria and the portability of the evaluated biochar production systems, localized biochar production closer to concrete manufacturing facilities may be feasible under suitable feedstock availability conditions. As an illustrative example, a localized transport scenario of approximately 12 km was evaluated based on the proximity between the Gresswell Forest Nature Conservation Reserve and a nearby concrete mixing plant. Under this scenario, biochar transport emissions were reduced to 0.19 kg CO_2_-eq, representing a 99.5% reduction compared to the Queensland transport case. Consequently, the total GWP of the BC32WR mix decreased by approximately 2.05%, highlighting the sensitivity of environmental performance to transport distance and the potential environmental benefits of localized biochar production.

Other contributors, such as diesel use in machinery, oil consumption, including heavy fuel oil and light fuel oil for heating, and lubricating oil for equipment maintenance, electricity, and waste treatment, had minimal impact on GWP, largely due to the use of low-voltage electricity in the production process. However, given that Victoria’s electricity grid is 87% fossil fuel-based, primarily from brown coal, emissions from electricity-dependent operations remain significant. Shifting to renewables like hydro, wind, or solar could substantially lower these emissions (Patrisia et al. 2022; Yu et al. 2017).

Previous LCA results indicate that biochar incorporation notably lowers CO_2_ emissions, and the combined use of supplementary cementitious materials (SCMs) can further enable carbon-negative concrete production (Chen et al. 2022; Azzi et al. 2019). A mix with 30 wt% biochar replacing recycled fine aggregates reduced total CO_2_ emissions by 86.2%, from 421 kg to just 58 kg CO_2_ per tonne of biochar block (Chen et al. 2022). Further replacing 30 wt% cement with SCMs, such as GGBFS, silica fume, PFA, or metakaolin, yielded a net negative carbon footprint, with GWP values ranging from −59 to −65 kg CO_2_ per tonne. Notably, the 30% metakaolin-biochar mix achieved a compressive strength 87% above the minimum 7 MPa requirement for bricks, while sequestering 59 kg CO_2_ per tonne, highlighting biochar’s dual contribution to performance and sustainability (Chen et al. 2022; Aman et al. 2022).

Figure S5 (Supplementary Information) illustrates that cement production is the dominant contributor to environmental impacts in both wood residue and plantation scenarios, with transportation as the second major contributor in most categories. The impact of biochar remains relatively minor across categories, except for GWP and photochemical oxidation.

#### Acidification potential

The acidification potential of CC32, BC32WR, and BC32WP was 1.2, 1.38, and 1.66 kg SO_2_-eq per m^3^ of concrete, respectively (Fig. 4). Cement production was the dominant contributor, accounting for 50–73% of total acidification impacts, consistent with previous studies (Patrisia et al. 2022). Transportation of biochar also contributed due to long transport distances. A detailed contribution analysis and sensitivity assessment of transport distance are provided in the Supplementary Information (Section S3.2).

#### Eutrophication potential

The eutrophication potential of the control concrete mix was 0.242 kg PO_4_^3^⁻-eq per m^3^ of concrete. The BC32 mix showed a 28.5% increase without plantation activities and a 165% increase when forestry operations were included. Cement production remained the major contributor, although the relative contribution from biochar increased when upstream forestry processes were considered. A detailed contribution analysis is provided in the Supplementary Information (Section S3.2). Detailed contribution breakdowns for additional environmental impact categories are provided in Fig. S5 in the Supplementary Information.

### Sensitivity analysis—effect of permanence factor on carbon sequestration

To assess the influence of biochar stability on long-term carbon sequestration, a sensitivity analysis was conducted using the permanence factor (PF) model developed by Puro.earth (puro.earth 2024). Although originally designed for soil systems, the model is conservatively applied to concrete, where biochar is expected to maintain similar stability. The PF estimates the fraction of organic (non-mineral) carbon in the biochar that remains after 100 years, based on its H/Cₒᵣ_g_ molar ratio and the regional temperature.

A constant temperature of 18.6 °C, representative of 10 m soil depth in Victoria, was applied across all scenarios (Narsilio et al. 2014). Based on this temperature, the coefficients used in the linear equation were *c *= 1.0184 and *m* = –0.6472, interpolated between the values provided in the methodology for 15 °C and 20 °C. The PF was calculated using the following equation:

4 $$PF=m\times \left(H/{C}_{org}\right)+c$$

$$PF=-0.6472\times \left(\frac{H}{{C}_{org}}\right)+1.0184$$

The sensitivity analysis included biochar produced from the four systems: 6050 Carbonator, ACB, OK, and BSI. Their respective fixed carbon contents, hydrogen percentages, H/C_mass_ ratios, and calculated permanence factors are presented in Table 7.

**Table 7:** Permanence factor and estimated sequestration potential based on H/Cₒᵣ_g_ values

System	Fixed C (%)	H (%)	H/C_mass_	PF	CO_2_ Sequestered (kg CO_2_-eq/kg biochar)	Reference
6050	85	1.14	0.0134	0.91	2.85	(Senadheera et al. 2023)
ACB	89	1.14	0.0128	0.92	3.00	(Senadheera et al. 2023)
OK	76	3.94	0.0518	0.62	1.72	(Rehrah et al. 2016)
BSI	65	5.40	0.0831	0.37	0.89	(Senadheera et al. 2023)

These results demonstrate that biochar with lower H/Cₒᵣ_g_ ratios, typically resulting from higher pyrolysis temperatures and longer residence times (Senadheera et al. 2023), has higher permanence and carbon sequestration potential. For example, biochar from the ACB system exhibits the highest PF (0.92), while BSI shows the lowest (0.37), underscoring the importance of the production method in determining long-term climate benefits.

A sensitivity analysis for the Tigercat 6050 biochar illustrates the influence of fixed carbon content on the net climate balance of BC32WR. When the fixed carbon content is reduced to 65%, the long-term carbon storage potential decreases to 0.89 kg CO_2_-eq/kg biochar, resulting in higher concrete net GWP values of 1512 kg CO_2_-eq m⁻^3^ (Recommendation method) and 467.9 kg CO_2_-eq m⁻^3^ (Carbon–Neutral method). Conversely, increasing the fixed carbon content to 90% enhances carbon storage and reduces the net GWP to 1287 and 243 kg CO_2_-eq m⁻^3^, respectively. These results demonstrate that the climate performance of BC32WR is highly sensitive to biochar quality, highlighting the importance of optimising production conditions to maximise stable carbon retention.

## Discussion

### Carbon accounting and sequestration dynamics

The definition of system boundaries and carbon accounting methods plays a pivotal role in the outcome of LCA. This study underscores that a clear distinction between biogenic and fossil carbon emissions is essential. Processes such as land-use change, biomass harvesting, and combustion significantly influence GWP and other impact categories, including acidification, eutrophication, freshwater ecotoxicity, particulate matter formation, and resource depletion.

Previous LCA studies on biochar systems commonly adopt either the carbon–neutral or recommendation-based biogenic carbon accounting approaches, both of which can indicate climate benefits depending on system boundaries and assumptions. In this study, both methods are intentionally applied to demonstrate how carbon accounting choices influence reported GWP outcomes. The Recommendation method treats biogenic CO_2_ emissions as real-time releases, whereas the Carbon–Neutral method assumes their offset within a sustainably managed carbon cycle. Presenting results under both frameworks enables a transparent comparison and highlights the sensitivity of climate impact results to biogenic carbon treatment, underscoring the importance of consistency and clarity in sustainability assessments of biochar-based construction materials.

Accordingly, interpretation of climate impacts depends strongly on the selected accounting framework and biomass sourcing assumptions. Under the Recommendation method, residual biomass scenarios may still exhibit positive production-stage GWP due to inclusion of biogenic CO_2_ emissions during pyrolysis, while plantation-based systems that include forest growth and biogenic CO_2_ uptake may achieve net-negative GWP outcomes. In contrast, the Carbon–Neutral method excludes biogenic CO_2_ flows from GWP calculations, thereby placing greater emphasis on fossil-derived emissions and stable carbon retained in biochar within the assessed system.

Although this study adopts a cradle-to-gate system boundary ending at concrete production, long-term carbon retention associated with stable biochar was evaluated separately through the net carbon balance assessment. The carbon storage benefit of biochar was considered at the point of concrete application, as stable biochar carbon becomes physically embedded within the cement matrix over long periods. Previous LCA studies (Rajabi Hamedani et al. 2019; Puettmann et al. 2020) adopted similar approaches by applying sequestration credit at the use stage even within shorter system boundaries. This treatment is also consistent with the Puro.earth carbon methodology, which permits carbon crediting upon the application of stable biochar. To maintain transparency, results were presented both with and without sequestration credit.

Under the Recommendation method, the observed positive GWP during biochar production reflects real-time CO_2_-equivalent emissions, often described as “carbon debt.” This concept refers to the temporary rise in atmospheric CO_2_ compared to baseline scenarios where biomass would decay naturally or remain unharvested. The length of this payback period depends on the baseline: for example, forest residues replacing coal can achieve payback in a few years, while harvesting whole trees to replace natural gas may take over 200 years (Bentsen 2017). The pyrolysis of forestry residues into biochar offers a viable strategy to reduce this carbon debt. Much of the carbon is stabilized and stored long-term, thereby contributing to lower net emissions. In sustainably managed forests, biogenic CO_2_ is part of a closed-loop cycle, where CO_2_ absorbed during growth is released during pyrolysis, contributing to a balanced biogenic carbon cycle (Puettmann et al. 2020; Sahoo et al. 2021; Forest and Wood Products Australia 2023). Non-CO_2_ biogenic emissions, such as CH_4_ and N_2_O from forest soils, residue storage, and combustion are included as biogenic GHG emissions other than CO_2_ (Forest and Wood Products Australia Ltd 2015).

### Environmental performance of biochar systems

Environmental impacts across the four pyrolysis systems studied varied significantly. Under the Recommendation method, the 6050 Carbonator exhibited the highest GWP, primarily due to low biochar yield (~7.5%), which increased feedstock demand and biogenic CO_2_ emissions. However, most of these emissions (91.2%) represent short-term releases of previously sequestered carbon rather than fossil-derived carbon. Additionally, there is a small portion of the total GWP that results from the biochar production process itself. Greenhouse gases such as N_2_O and CH_4_, released during pyrolysis, contributed approximately 8% of the total GWP. Supporting operations, such as skidding and diesel combustion, further added around 0.0832 kg CO_2_-eq, accounting for less than 0.8% of the overall GWP.

In contrast, when assessed using the Carbon–Neutral method, which has been commonly employed in previous LCA studies (Puettmann et al. 2020), the 6050 system demonstrated the lowest GWP, outperforming the ACB, OK, and BSI systems. The favourable performance of the 6050 highlights the importance of crediting stable carbon storage when biochar is used in long-lived applications. Conversely, the BSI method consistently exhibited the highest environmental impacts across most categories due to reliance on diesel-based electricity, energy-intensive feedstock preprocessing, and higher propane use, nearly four times that of the ACB system. Similarly, the OK exhibited elevated impacts, with 2.3 times higher propane consumption and material preparation needs compared to ACB.

Despite higher GWP under the Recommendation method, the 6050-system performed best in several impact categories, including abiotic depletion, particulate matter formation, and ionizing radiation. In contrast, the BSI system exhibited the worst performance in most categories, except for freshwater ecotoxicity, where the 6050-system ranked highest due to increased feedstock requirements. In this case, over 99% of ecotoxicity stemmed from plantation and harvesting activities, reinforcing the importance of biochar yield efficiency and sustainable upstream practices.

Utilizing forest residues for biochar production avoids the more harmful emissions that would occur through decay or open burning. For instance, the anaerobic decomposition of 1 kg of forest residue emits approximately 0.672 kg CO_2_-eq from CH_4_ and an additional 0.147 kg CO_2_-eq through natural decay. The 2019 bushfires in Australia, which burned around 7.4 million hectares, released an estimated 830 million tonnes of CO_2_-eq, equivalent to 166% of the nation’s annual GHG emissions (Forest and Wood Products Australia 2023).

Redirecting such biomass into value-added products like biochar may provide significant climate mitigation potential. Empirical data support this approach: pile burning emits around 1.8 kg CO_2_-eq per kg of residue, whereas the 6050 system emits just ~0.81(10.8/13.33) kg CO_2_-eq/kg residue under the Recommendation method. Moreover, Puettmann et al. (2020) report that biochar systems can achieve net GWP values as low as 0.10–1.63 kg CO_2_-eq/kg of feedstock, representing up to a 40-fold reduction compared to open burning.

### Integration of biochar in concrete applications

The integration of biochar into concrete shows both potential benefits and important limitations from a climate impact perspective. Under the Recommendation method, BC32WR exhibits a higher GWP than conventional concrete, primarily due to the immediate inclusion of biogenic CO_2_ emissions from biochar production. However, the results also demonstrate that overall climate performance depends strongly on biomass source, production technology, transport distance, and carbon accounting assumptions. In contrast, the Carbon–Neutral method places greater emphasis on long-term carbon retention benefits, with BC32WR showing an approximately 40% lower net impact than conventional concrete once sequestration is considered. These findings indicate that biochar incorporation does not automatically reduce GWP and may increase short-term impacts depending on system boundaries and modelling assumptions. From a policy perspective, incentives or credits for biochar-based construction materials should be conditional on clearly defined system boundaries, verified carbon storage assumptions, and transparent biogenic carbon accounting to avoid misleading climate benefit claims.

Moreover, substituting biochar for sand contributes to resource conservation. While sand production emits only ~0.0040 kg CO_2_-eq per kg, milled biochar production emits 11.97 kg CO_2_-eq under the Recommendation method and 1.06 kg CO_2_-eq under the Carbon–Neutral method. Despite higher energy requirements, biochar showed lower impacts than sand in categories such as ODP, human toxicity, and water scarcity, due to its waste-derived origin and carbon storage capacity.

Results showed moderately higher impacts in other environmental categories in addition to the GWPs compared to control concrete. Specifically, BC32WR gave increases in abiotic depletion (21%), fossil fuel use (15%), acidification (15%), ozone depletion (36%), human toxicity (22%), freshwater ecotoxicity (9%), and ionizing radiation (5%). However, in all these categories, biochar production remained below 4.5%. Cement production and transportation were identified as the primary impact drivers.

Transport-related emissions also contributed notably to the environmental performance of the BC32WR mix. Long-distance transport contributed substantially to impact categories such as photochemical oxidation (57% from biochar production and 8% from transport) and particulate matter formation (13% from biochar and 9% from transport).

The long-term carbon storage potential of biochar depends on its stability, often measured by H/Cₒᵣ_g_ ratios and fixed carbon content. Low H/Cₒᵣ_g_ and high fixed carbon content indicate good permanence. Biochar produced by the ACB and 6050 systems, which operate under relatively higher pyrolysis temperatures, may therefore provide greater long-term carbon retention potential.

A sensitivity analysis showed that increasing fixed carbon content from 65 to 90% reduced the net GWP of BC32WR by 15% under the Recommendation method and by 48% under the Carbon–Neutral method. These findings underscore the need to optimize pyrolysis conditions, particularly temperature and residence time, to maximize environmental benefits while maintaining energy efficiency.

### Logistics, circularity, and broader sustainability impacts

In parallel, the study emphasizes the critical role of transportation in LCA, particularly for distributed biochar production. Transport distance, mode, and fuel type, along with feedstock location and availability, significantly influence emissions across multiple impact categories (Patrisia et al. 2022, 2025b; Rosado et al. 2022). For instance, road transport per tonne-kilometre (tkm) contributes approximately 0.168 kg CO_2_-eq, 7.3 × 10⁻^4^ kg SO_2_-eq, 1.6 × 10⁻^4^ kg PO_4_^3^⁻-eq, and 1.27 × 10⁻^9^ CTUh to GWP, acidification, eutrophication, and human toxicity (cancer), respectively. Consequently, transportation logistics can substantially influence the environmental outcomes of biochar-integrated concrete systems.

The approximately 2000 km Queensland-to-Victoria biochar transport scenario considered in this study imposed a notable environmental burden within the BC32WR system. However, the portable and mobile nature of systems such as the 6050 Carbonator may provide flexibility for decentralized biochar production closer to biomass sources and concrete manufacturing facilities. An illustrative localized transport scenario was evaluated in which biochar transport distance was reduced to approximately 12 km. Under this scenario, transport-related emissions were reduced by approximately 99.5%, resulting in a 2.05% reduction in the total GWP of the BC32WR concrete system. The evaluated interstate (~2000 km) and localized (~12 km) transport scenarios represent contrasting boundary conditions intended to illustrate the sensitivity of environmental performance to transport distance. In practice, environmental impacts would likely vary within this range depending on regional biomass availability, transport infrastructure, and proximity to concrete production facilities.

Alternative transport modes also present opportunities for improvement. Rail transport, for instance, has been shown to reduce CO_2_ emissions by up to 25% compared to road transport (Shobeiri et al. 2021). Therefore, optimizing logistics through decentralized production and efficient transport can further enhance the environmental performance of biochar-based construction.

Beyond transportation considerations, biochar presents a promising pathway for advancing sustainable construction within a circular economy framework. In Australia, around 2 million tonnes of forestry residues are generated annually in New South Wales (NSW Department of Primary Industries n.d.), providing a substantial feedstock for biochar production. Converting this biomass into biochar for use in concrete enables both emissions reduction and waste valorization, contributing to more sustainable construction practices. Furthermore, integrating biochar utilization with afforestation and managed plantation strategies, such as Radiata pine cycles in Australia (Azzi et al. 2022; Forest and Wood Products Australia 2023) and short-rotation forestry models globally can enhance long-term carbon sequestration.

Unlike direct biomass combustion pathways, biochar production retains a portion of biogenic carbon in stable solid form, enabling long-term carbon storage within concrete materials. Therefore, the use of biochar in construction applications provides not only material substitution benefits but also an opportunity for carbon retention and waste valorization within the built environment. By bridging rural biomass resources with urban construction demands, biochar facilitates the development of closed-loop, low-carbon material systems. This integration supports broader climate goals and promotes sustainable land and resource management.

### Limitations and future research

This study provides a comparative environmental assessment of forestry biochar systems and biochar-integrated concrete; however, several limitations should be acknowledged.

The transport relocation scenario evaluated in this study was intended as an illustrative sensitivity analysis rather than a comprehensive regional feasibility assessment. Practical implementation depends on forestry residue availability, transport infrastructure, operational scale, and proximity to concrete production facilities. Future studies should therefore incorporate GIS-based spatial and logistics analysis.

In addition, the BC32 concrete mixtures were based on previously reported biochar replacement performance, and experimentally validated compressive strength data for the specific mixtures were not included. Future work should integrate experimentally measured mechanical performance and strength-normalized functional units for performance-equivalent environmental comparison.

Furthermore, direct land-use change emissions and alternative forestry residue utilization pathways, such as bioenergy generation, were outside the scope of the present study and should be further investigated in future research.

## Conclusion

This study employed LCA to evaluate concrete mixes incorporating timber-based biochar, produced through different pyrolysis methods, as a partial replacement for sand. Based on the environmental performance across multiple impact categories, carbon accounting, and the influence of biochar production processes, the following conclusions can be drawn:

1. Among the four biochar production methods assessed, the 6050 Carbonator demonstrated the lowest GWP under the Carbon–Neutral assumption, outperforming the OK, ACB, and BSI systems. However, when biogenic CO_2_ emissions are fully accounted for under the Recommendation method, the 6050-system had a higher GWP than both the OK and ACB methods, primarily due to lower biochar yield, which results in increased release of biogenic gases per unit of biochar produced.
2. Biochar quality, particularly carbon stability, plays a critical role in enhancing environmental performance. Increasing the fixed carbon content from 65% to 90% boosted the carbon sequestration potential by 237%, resulting in a reduction in net GWP impact 15% under the Recommendation method and 48% under the Carbon–Neutral method. These results emphasize the benefits of having optimized pyrolysis conditions, such as temperature and residence time, to produce stable biochar with low H/Cₒᵣ_g_ ratios and high fixed carbon content for long-term climate benefits.
3. When 15% of sand was replaced with biochar derived from wood residues, the concrete mix (BC32WR) achieved a 40% reduction in net impact in GWP for the Carbon–Neutral method, including the benefit of carbon sequestration. However, several other environmental impact categories showed increases of up to 35% compared to the control mix. These included AP, EP, particulate matter formation, HTP, freshwater ecotoxicity, ionizing radiation, ADP, and ODP. Importantly, the biochar itself contributed less than 13% to most of these increases, while transportation, especially long-distance delivery, was responsible for up to 77% of the added burden. As biochar production equipment is portable, relocating production closer to concrete manufacturing sites offers a potential strategy to significantly reduce these transport-related impacts.
4. BC32WP concrete exhibits a negative GWP (−17.49 kg CO_2_ -eq./m^3^) under the Recommendation method, indicating a net reduction of atmospheric CO_2_ even without explicitly crediting biochar carbon sequestration. However, the inclusion of upstream plantation and harvesting activities increases impacts in some non-GWP categories, while improvements are observed in others, such as water scarcity.
5. According to the impact and benefit assessment, producing biochar from sustainably managed forests, short-rotation plantations, or wood residues and incorporating it into concrete may provide significant environmental and socio-economic benefits, such as reducing carbon emissions and waste, mitigating wildfire risk by managing forest residues, and promoting the circular use of forestry by-products, while supporting climate-positive construction.
6. This study further demonstrates that assumptions regarding biomass origin and biogenic carbon accounting strongly influence the reported climate impacts of biochar-based concrete. The choice between carbon-neutral and recommendation approaches can lead to contrasting GWP outcomes, highlighting that methodological decisions are as important as material choices when informing policy, design, and investment decisions for low-carbon construction.

## Supplementary Information

Below is the link to the electronic supplementary material.

(DOCX 1.72 MB)

## Data Availability

All data generated or analyzed during this study are included in this published article and its Supplementary Information. Additional data are available from the corresponding author upon reasonable request.
