# Supplementary material for: Environmental life cycle assessment of forestry biochar for low-carbon concrete: influence of biogenic carbon accounting
Source: Environ Sci Pollut Res Int. 2026 Nov 4;33(40):18169–88. doi: 10.1007/s11356-026-38335-1 (PMC13769716; doi:10.1007/s11356-026-38335-1)
Supplement: Supplementary file 1 [file 11356_2026_38335_MOESM1_ESM.docx]

# Environmental Life Cycle Assessment of Forestry Biochar for Low-Carbon Concrete: Influence of Biogenic Carbon Accounting

Environmental Science and Pollution Research

^1^Supun Karunarathna, ^1^Chamila Gunasekara*, ^1^David Law, ^1^Yulin Patrisia, ^2^Letiscia Xavier, ^1^Sujeeva Setunge,

1)Civil & Infrastructure Engineering, School of Engineering, RMIT University, Melbourne, Victoria 3000, Australia

2)Vital Chemical, 170 Bellwood St, Darra, Queensland 4076, Australia

*Corresponding author: chamila.gunasekara@rmit.edu.au

# S1 Biochar production technologies

## S1.1 6050 Carbonator Method

The Tigercat 6050 is a self-powered, track-driven mobile pyrolysis unit designed for on-site carbonisation of wood debris (Fig. S1). The system utilises flame-cap high-temperature pyrolysis to convert biomass, such as logs, limbs, stumps, and brush, into biochar, operating at approximately 820–1370°C depending on combustion conditions. Based on operational data obtained from industry sources, the system can process approximately 20 tonnes of woody biomass per hour. The system has dimensions of approximately 12.19 m in length, 3.61 m in width, and 3.66 m in height. A key advantage of the 6050 Carbonator is that it does not require feedstock preprocessing; unprocessed or processed clean wood and vegetative debris can be directly fed into the system without chipping, grinding, or drying. The feedstock acts as the fuel source, and both over-chamber and under-chamber airflow are provided to ensure efficient pyrolysis while preventing complete oxidation to ash.

Fig. S1 Tigercat 6050 Carbonator

The system design features a robust thermal ceramic chamber that supports stable high-temperature operation. As the material carbonizes, the biochar falls through a grate and is quenched and discharged via an integrated auger system. This process significantly reduces the volume of wood waste, by up to 90%, and eliminates the need to transport or dispose of residuals off-site. In addition to producing a usable carbon-rich biochar product, the 6050 Carbonator is suitable for use in remote forest locations, post-disaster cleanup areas, and mill sites. Operation is relatively low-cost and efficient, requiring only a single operator, and it offers built-in safety features for handling and cooling the char.

## S1.2 Air Curtain Burner (ACB) Method

ACB is a high-capacity, refractory-lined combustion system designed for the on-site thermal treatment of woody biomass (Fig. S2). In this study, the pyrolysis process follows methodologies described in the literature (Sahoo et al. 2021; Puettmann et al. 2020). The S-220 model ACB has a 36-kW engine to operate a blower. Functioning as a batch-operated system, the S-220 is a scaled-up version of the Oregon Kiln, offering higher throughput and elevated combustion temperatures due to robust insulation. Operating temperatures of approximately 680–750°C for flame-curtain systems such as the ACB and Oregon Kiln, with the ACB generally operating at comparatively higher temperatures and carbonization intensity(Puettmann et al. 2020) .

Fig. S2 Air Curtain Burner (Lee and Han 2017)

The process commences with the placement of the unit on flat, cleared ground (Lee and Han 2017). Biomass is loaded into the chamber using a skidder, a typical in-field loading method for small-scale mobile units. The skidder efficiency depends on the distance to the feedstock and the level of material sorting required. During pyrolysis mode, adopted in this study, the blower is assumed to remain off to limit oxygen exposure and promote carbonization. In contrast, when used in incineration mode, the blower operates continuously to enable complete combustion of the ash (Sahoo et al. 2021).

Following ignition, biomass is gradually added until the chamber reaches capacity. Water is not poured directly into the burn chamber to prevent thermal shock, as this could damage the refractory lining. Instead, the ACB unit is lifted or dragged forward, allowing the hot biochar to discharge from the open bottom (Puettmann et al. 2020). The biochar is then quenched with water and spread out using a skid steer loader to cool (Sahoo et al. 2021; Puettmann et al. 2020). Compared to the Oregon Kiln, the ACB yields biochar with a higher fixed carbon content, owing to its ability to operate at elevated temperatures.

## S1.3 Oregon Kiln (OK) Method

Cleft timber feedstock is transported to the biochar production facility where the material undergoes further size reduction using a chainsaw to improve stacking and burning efficiency. The processed biomass is then mechanically loaded into the OK using a skidder. The OK is a portable, low-cost flame-cap pyrolysis unit optimized for in-forest biochar production as an alternative to open-pile burning (Sahoo et al. 2021) (Fig. S3). The kiln has a truncated pyramidal shape, fabricated with 2 mm thick mild steel, with standard dimensions 1.5 m² top base, 1.2 m² bottom base, and 0.6 m height, providing a capacity of 1.1 m³ (Sahoo et al. 2021), although there are different dimensions in which kilns can be found (Cornelissen et al. 2016).

Fig. S3 Oregon Kiln (Sahoo et al. 2021)

The OK operates on the principle of flame carbonization, where a flame curtain on top of the biomass layer restricts oxygen ingress, enabling pyrolysis while simultaneously combusting volatile gases (Puettmann et al. 2020; Cornelissen et al. 2016). The process starts by kindling a fire at the base. Once a glowing char bed is formed, fresh biomass is gradually added in layers. Each new layer ignites, creating a flame cap that excludes air from the lower layer and ensures efficient pyrolysis. As a result, smoke emissions are minimal, as most volatiles are burned within the flame zone. When the kiln reaches capacity, the biochar is quenched using water or sealed with a dirt cap to prevent further oxidation and preserve carbon content (Cornelissen et al. 2016; Sahoo et al. 2021).

Based on the design of Japan’s Smokeless Kiln, the OK produces biochar with conversion efficiencies ranging from 13% to 20%, depending on the feedstock and process parameters (Puettmann et al. 2020). The design and function are similar to the Kiln Kon-Tiki. This conical, top-lit updraft gasifier is highly portable (approx. 80 kg in weight) and suitable for on-site biochar production from untreated wood waste (Zaramí 2021). Like Kon-Tiki, the OK can operate without chipping or pelletizing the feedstock, making it ideal for processing uncomminuted woody biomass such as branches and cleft timber.

## S1.4 Biochar Solutions Incorporated (BSI) Method

The BSI system is a mobile down-draft gasifier for continuous biochar production from chipped or ground woody biomass (Fig. S4). In this study, the BSI system is operated at a near-forest location using a diesel generator to supply the required 20 kW of electrical power (Puettmann et al. 2020). The gasification process occurs under oxygen-limited conditions, with biochar formed as the biomass moves through the heated reactor chamber. Compared with high-temperature flame-curtain pyrolysis systems, the BSI process operates under comparatively lower carbonization severity, contributing to higher biochar yields but lower fixed carbon content.

The unit operates continuously, and the biochar is discharged through a cooled auger system into sealed containers (Puettmann et al. 2020). Compared to batch-based technologies, the BSI system enables consistent production, and its mobility supports decentralized biochar generation near the biomass source. Using a diesel generator at the forest site allows off-grid deployment, though it contributes to fossil carbon emissions that must be considered in life cycle assessments.

Fig. S4 Biochar Solutions Incorporated (Biochar Solutions Inc. 2025)

This study assumes that the wood residues consist of 2/3 bole wood and 1/3 tops and branches. During feedstock preparation for the BSI system, the logs (bole wood) undergo sorting and chipping, while the tops and branches are processed through grinding. Both fractions are then screened to achieve a uniform particle size. The screened biomass is subsequently loaded into the BSI biochar production system. The system has the potential to operate either on grid electricity or on electricity generated onsite, such as through Power Pallets, diesel generators, or gasifier units (Sahoo et al. 2021; Puettmann et al. 2020).

# S2 Life cycle inventory data

Table S1 Inventory for LCA study

| Data type | Source |
| --- | --- |
| Biochar production | |
| Tigercat 6050 carbonator | Manufacturer technical documentation (Tigercat International Inc. 2020) |
| Air curtain burner (ACB) | (Sahoo et al. 2021; Puettmann et al. 2020) |
| Oregon Kiln (OK) | (Sahoo et al. 2021; Puettmann et al. 2020) |
| Biochar Systems Incorporated (BSI) | (Puettmann et al. 2020; Sahoo et al. 2021; Oneil et al. 2017) |
| Biochar emission factors | (Sahoo et al. 2021; Puettmann et al. 2020; Tigercat International Inc. 2020) |
| Concrete production | |
| OPC, Australian Average | AusLCI |
| Gravel, crushed, at mine | Ecoinvent, AusLCI |
| Sand, at mine | Ecoinvent, AusLCI |
| Melamine formaldehyde resin, at the plant | Ecoinvent, AusLCI |
| Disposal of concrete, 5% water, to inert material landfill | Ecoinvent |
| Tap water, at user, Victoria | AusLCI |
| Concrete mixing plant | Ecoinvent, AusLCI |
| Electricity, low voltage | AusLCI |
| Natural gas burned in industrial furnace | Ecoinvent, AusLCI |
| Heavy fuel oil, burned in industrial furnace | Ecoinvent, AusLCI |
| Light fuel oil, burned in industrial furnace | Ecoinvent, AusLCI |
| Diesel burned in building machine | AusLCI |
| Propane burned in building machine | Ecoinvent, AusLCI |
| Transport truck, 16–28, fleet average | AusLCI |
| Skidder | Ecoinvent, AusLCI |
| Diesel, burned in agricultural machinery | Ecoinvent, AusLCI |
| Power sawing, without a catalytic converter | Ecoinvent, AusLCI |

Table S2 Feedstock preparation and transportation data for the BSI system (Sahoo et al. 2021)

| Equipment | Fuel consumption rate  (L/tonne) | Used for |
| --- | --- | --- |
| John Deere 2954D Loader | 0.35 | Sorting Logs |
| John Deere 2954D Loader | 0.71 | Loading logs |
| 765 HP Peterson Pacific model 4300 Micro-chipper | 2.55 | Chipping (Boles Only) |
| 1050 HP Peterson Pacific horizontal grinder | 2.99 | Grinding (Tops Only) |
| Peterson Pacific Deck screener | 1.6 | Screening |
| Tractor | 0.29 | Tractor Transport (Staging Site) |

Table S3 After the biochar production process (milling process)

| Activity | Item | Value |
| --- | --- | --- |
| Loading the biochar | Loading capacity | 1 m^3^/min |
|  | Fuel consumption | 6 L/h |
| Unloading | Unloading capacity | 11.2 tonnes/hr |
|  | Fuel consumption | 6 L/h |
| Loading into milling equipment | Loading rate | 11.2 tonnes/hr |
|  | Fuel consumption | 6 L/h |
| Milling | Electricity consumption | 29.75 kW |
| Loading the biochar | Loading rate | 35 bags/day |
|  | Fuel consumption | 40 l/week |

Table S4 Parameters applied to estimate GHG emissions due to wood residue decay

| Parameter | Value | Description / Justification |
| --- | --- | --- |
| DOC (Degradable Organic Carbon) | 0.4 | Default value for wood waste based on IPCC/NIR methodology |
| DOC_f_ (Fraction of DOC that degrades) | 0.1 | Conservative degradation fraction for wood residues |
| MCF (Methane Correction Factor) | 1.0 | Assumes anaerobic decomposition conditions |
| F (Methane fraction of gas) | 0.5 | Standard IPCC assumption |
| OF (Oxidation Factor) | 0.1 | Portion of CH₄ oxidized before release |
| GWP100 for CH₄ | 28 | IPCC AR5 characterization factor |
| Molecular weight ratios | CH₄/C = 16/12, CO₂/C = 44/12 | Standard molecular conversion |

# S3 Additional environmental impact indicators

## S3.1 Biochar production

## S3.1.1 Abiotic Depletion Potential (Fossil Fuels)

ADP quantifies the use of non-renewable energy resources, primarily diesel and propane consumption during biochar production (Renouf 2016). Among the four systems assessed, BSI gave the highest depletion, at 2.92 MJ NCV, due to its reliance on diesel-powered electricity generation, intensive feedstock preprocessing, and propane ignition. This was followed by OK with 1.64 MJ NCV, primarily attributed to propane consumption. The ACB exhibited a lower impact of 0.715 MJ NCV. The Tigercat 6050 Carbonator demonstrated the lowest fossil fuel depletion at 0.0686 MJ NCV, reflecting minimal energy input and operational efficiency. When wood growth and harvesting stages are included, abiotic depletion increases by approximately 0.03905 MJ NCV per kg of feedstock. The 6050 Carbonator remains the most energy-efficient overall. This variation further illustrates variability based on feedstock type and pretreatment requirements. For instance, slow pyrolysis of pig manure and willow wood chips has been reported to yield 2.106 MJ/kg and –15.08 MJ/kg biochar (Rajabi Hamedani et al. 2019).

## S3.1.2 Particulate Matter (PM_2.5_)

PM emissions, a key indicator of air pollution and related health risks, varied significantly. The BSI system recorded the highest PM₂.₅ emissions, 3.14× 10⁻³ kgPM_2.5‐eq_, which is over 29 times higher than the Tigercat 6050 Carbonator, which had the lowest emissions. This elevated value in BSI is likely attributed to its greater reliance on diesel and propane and more intensive feedstock processing. The OK and ACB reported intermediate PM₂._5_ levels of 0.71 and 0.57 times the BSI, respectively.

**S3.1.3 Ozone Layer Depletion**

ODP refers to the potential of emissions, particularly chlorinated compounds, to degrade the stratospheric ozone layer, which protects life on Earth from harmful ultraviolet radiation (United States Environmental Protection Agency 2025). Among the four systems, the BSI had the highest ODP impact at 8.7 × 10⁻^10^ kg CFC-11 eq, followed by the OK (4.43 × 10⁻^10^) and ACB (1.94 × 10⁻^10^). The 6050 Carbonator had the lowest ODP value at 3.55× 10⁻^11^, indicating minimal contribution to ozone depletion. The elevated ODP values for BSI and OK are primarily attributed to their greater use of fossil-based fuels such as diesel and propane, which can emit ozone-depleting substances during combustion.

S3.2 Biochar concrete production
S3.2.1 Acidification potential

As shown in Table 6, the acidification potential of CC32, BC32WR, and BC32WP is 1.2, 1.38, and 1.66 kg SO₂-eq per m³ of concrete, respectively. Overall, cement production remains the dominant contributor, accounting for 50% to 73% of the total acidification impact. These findings are consistent with previous studies, which reported that approximately 75% of acidification impact in on-site concrete mixing is attributable to cement manufacturing, followed by transportation, contributing around 15% (Patrisia et al. 2022).

This is particularly evident for CC32, with a contribution of 0.257 kg SO₂-eq. For BC32, the transport of biochar from Queensland to the mixing site in Victoria adds 0.145 kg SO₂-eq, due to the long transportation distance and associated diesel emissions. A sensitivity analysis was conducted to assess the impact of relocating biochar production closer to the mixing facility. Transporting one tonne of biochar per km generates approximately 7.34 × 10^-4^ kg SO₂-eq. Relocating biochar production to a nearby forest 12 km from the mixing site reduces the acidification impact of transport by 99.5%, lowering it to just 8.43 × 10⁻^3^ kg SO₂-eq and improving the overall profile of the BC32 mix.

While the energy demand for concrete mixing has minimal influence on acidification potential (Patrisia et al. 2022), the impact of material processing and transport is more pronounced. On a per-kilogram basis, sand contributes 8.66 × 10⁻⁶ kg SO₂-eq, whereas biochar production contributes 5.5 × 10⁻⁴ kg SO₂-eq, primarily due to diesel usage in pyrolysis systems.

## S3.2.2 Eutrophication potential

The eutrophication potential of the control concrete mix was 0.242 kg PO₄³⁻-eq per m³ of concrete. The BC32 mix showed a 28.5% increase without including the plantation phase and a 165% increase when the wood plantation and harvesting stages were included. This substantial rise emphasizes the significant contribution of forestry operations to nutrient-related environmental burdens.

In all mixes, cement production remained the primary contributor to eutrophication, accounting for 67% in CC32 and decreasing to 52% and 25% in BC32WR and BC32WP, respectively. Conversely, the contribution from biochar increased considerably, from 12.7% (BC32WR) to 57.7% (BC32WP), an indication of the influence of upstream land-use and processing emissions.

The eutrophication impact from wood plantation and harvesting was calculated to be approximately 2.34 × 10⁻⁴ kg PO₄³⁻-eq per kg of harvested wood, highlighting its dominant role compared to the relatively minor contributions from milling and handling activities, which amounted to 1.26 × 10⁻⁵ kg PO₄³⁻-eq per kg of milled biochar. Transportation also played a notable role, contributing 0.0571 kg PO₄³⁻-eq in the CC32 mix and 0.087 kg PO₄³⁻-eq in the BC32 mix. Within BC32, biochar transport alone accounted for 0.032 kg PO₄³⁻-eq, due to the long distance of transportation.

**Figures**

|  |  |
| --- | --- |
|  |  |
|  |  |
|  | |

Fig. S5 Contribution of material and process stages to non-GWP environmental impact categories for CC32 and BC32 concrete mixes
